# Supplementary material for: Ribosomes slide on lysine-encoding homopolymeric A stretches
Source: eLife. 2015 Feb 19;4:e05534. doi: 10.7554/eLife.05534 (PMC4363877; doi:10.7554/eLife.05534)
Supplement: Supplementary file 1. — Primary sequence of mCherry with out of frame stop-codons highlighted. The nucleotide sequence of the Thrdx-HA-mCherry reporters (Figure 1A) with all out of frame stop codons after the insertion site highlighted in yellow. DOI: http://dx.doi.org/10.7554/eLife.05534.021 [file elife05534s001.docx]

**Supplementary File 1**

ATGGGATCTGATAAAATTATTCATCTGACTGATGATTCTTTTGATACTGATGTACTTAAGGCAGA TGGTGCAATCCTGGTTGATTTCTGGGCACACTGGTGCGGTCCGTGCAAAATGATCGCTCCGATT CTGGATGAAATCGCTGACGAATATCAGGGCAAACTGACCGTTGCAAAACTGAACATCGATCACA ACCCGGGCACTGCGCCGAAATATGGCATCCGTGGTATCCCGACTCTGCTGCTGTTCAAAAACGG TGAAGTGGCGGCAACCAAAGTGGGTGCACTGTCTAAAGGTCAGTTGAAAGAGTTCCTCGACGC TAACCTGGCCGGCTCTGGATCCGGTGATGACGATGACAAGCTGGGAATTATCACAAGTTTGTAC AAAAAAGCAGGCTCCGCGGCCGCCCCCTTCCACATGGGCTACCCATACGATGTTCCAGATTACG CTGCGGCCTACCCGTATGACGTACCGGATTATGCA(**INSERT**)GTGAGCAAGGGCGAGGAGGATA ACATGGCCATCATCAAGGAGTTCATGCGCTTCAAGGTGCACATGGAGGGCTCCGTGAACGGCC ACGAGTTCGAGATCGAGGGCGAGGGCGAGGGCCGCCCCTACGAGGGCACCCAGACCGCCAAG CTGAAGGTGACCAAGGGTGGCCCCCTGCCCTTCGCCTGGGACATCCTGTCCCCTCAGTTCATGT ACGGCTCCAAGGCCTACGTGAAGCACCCCGCCGACATCCCCGACTACTTGAAGCTGTCCTTCCC CGAGGGCTTCAAGTGGGAGCGCGTGATGAACTTCGAGGACGGCGGCGTGGTGACCGTGACCC AGGACTCCTCCCTGCAGGACGGCGAGTTCATCTACAAGGTGAAGCTGCGCGGCACCAACTTCCC CTCCGACGGCCCCGTAATGCAGAAGAAGACCATGGGCTGGGAGGCCTCCTCCGAGCGGATGTA CCCCGAGGACGGCGCCCTGAAGGGCGAGATCAAGCAGAGGCTGAAGCTGAAGGACGGCGGCC ACTACGACGCTGAGGTCAAGACCACCTACAAGGCCAAGAAGCCCGTGCAGCTGCCCGGCGCCT ACAACGTCAACATCAAGTTGGACATCACCTCCCACAACGAGGACTACACCATCGTGGAACAGTA CGAACGCGCCGAGGGCCGCCACTCCACCGGCGGCATGGACGAGCTGTACAAGTAA
